# Supplementary material for: Predicting the Glycemic Index of Biscuits Using Static In Vitro Digestion Protocols
Source: Foods. 2023 Jan 14;12(2):404. doi: 10.3390/foods12020404 (PMC9858452; doi:10.3390/foods12020404)
Supplement: Supplementary file 1 [file foods-12-00404-s001.zip › foods-2045113-supplementary.pdf]

Supplementary material

Table S1. Chemical compositions, and the GI and GL values of biscuits samples.

Sample ID	GI values	GL values	Moisture content [1]	Protein content [2]	Fat content [2]	Total carbohydrates	Total starch (%, db) [1]
9	52	35.36	4.20±0.52^{a}	7.50±1.18^{c}	15.30±2.74^{ab}	68.15±4.24^{a}	34.76±2.45^{a}
10	53	33.93	3.70±0.48^{b}	7.21±1.31^{c}	17.24±1.98^{a}	64.52±3.59^{a}	39.03±0.57^{a}
11	44	29.04	4.12±0.74^{a}	7.80±1.25^{c}	14.51±1.81^{b}	66.11±4.07^{a}	29.33±3.79^{b}
12	30	10.2	3.97±0.19^{ab}	29.55±2.35^{b}	8.83±1.14^{c}	34.12±3.67^{b}	17.13±1.82^{c}
13	28	8.57	2.15±0.39^{d}	32.17±1.93^{a}	11.39±1.52^{c}	30.67±2.98^{b}	14.32±1.57^{c}
14	19	6.27	3.56±0.81^{c}	27.64±1.72^{b}	10.80±1.77^{c}	33.04±4.52^{b}	15.65±2.73^{c}

[1] Data was based on triplicate measurements, [2] data was based on duplicate measurements. Values with different letters in the same column are significantly different at $p < 0.05$.

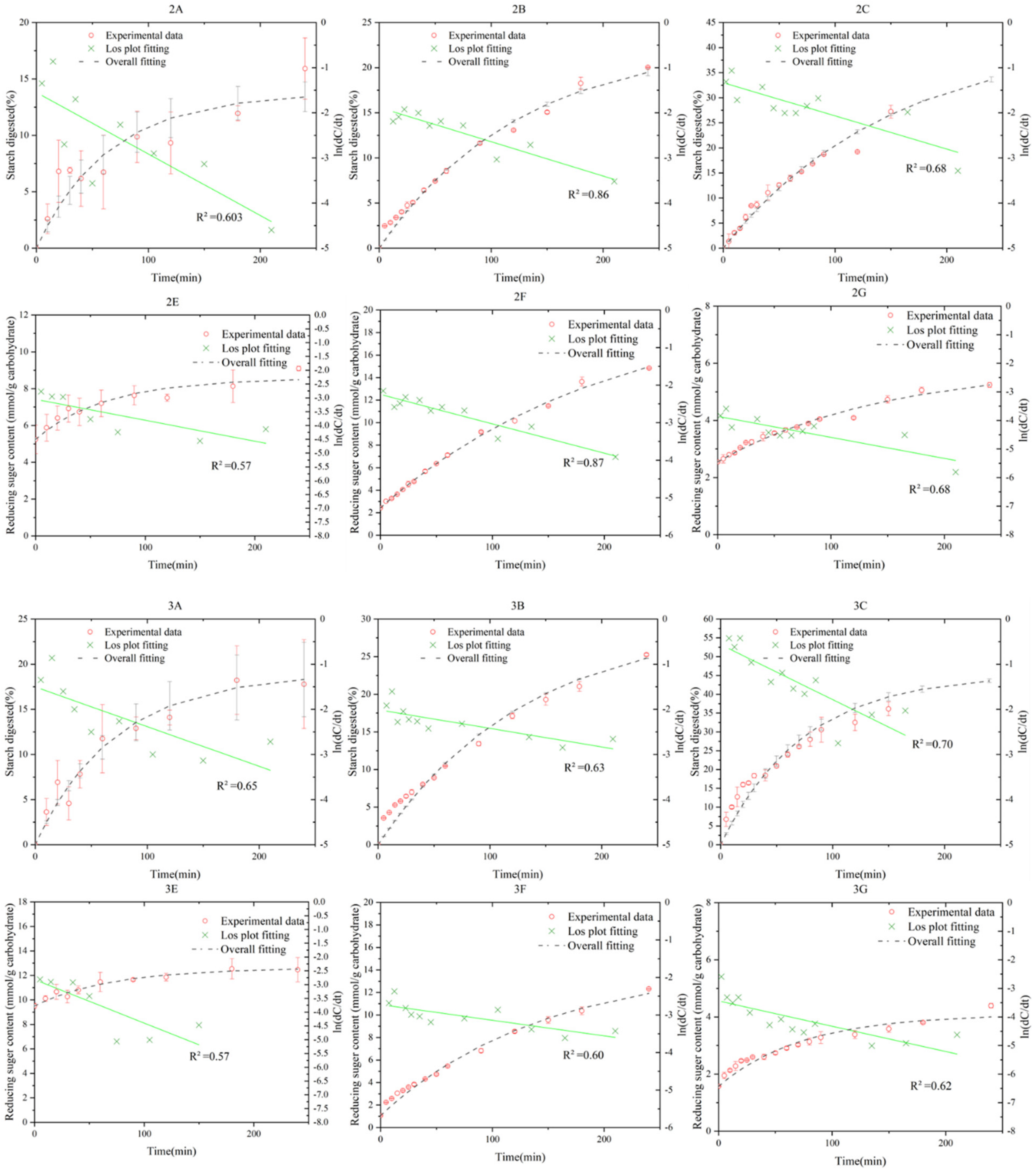

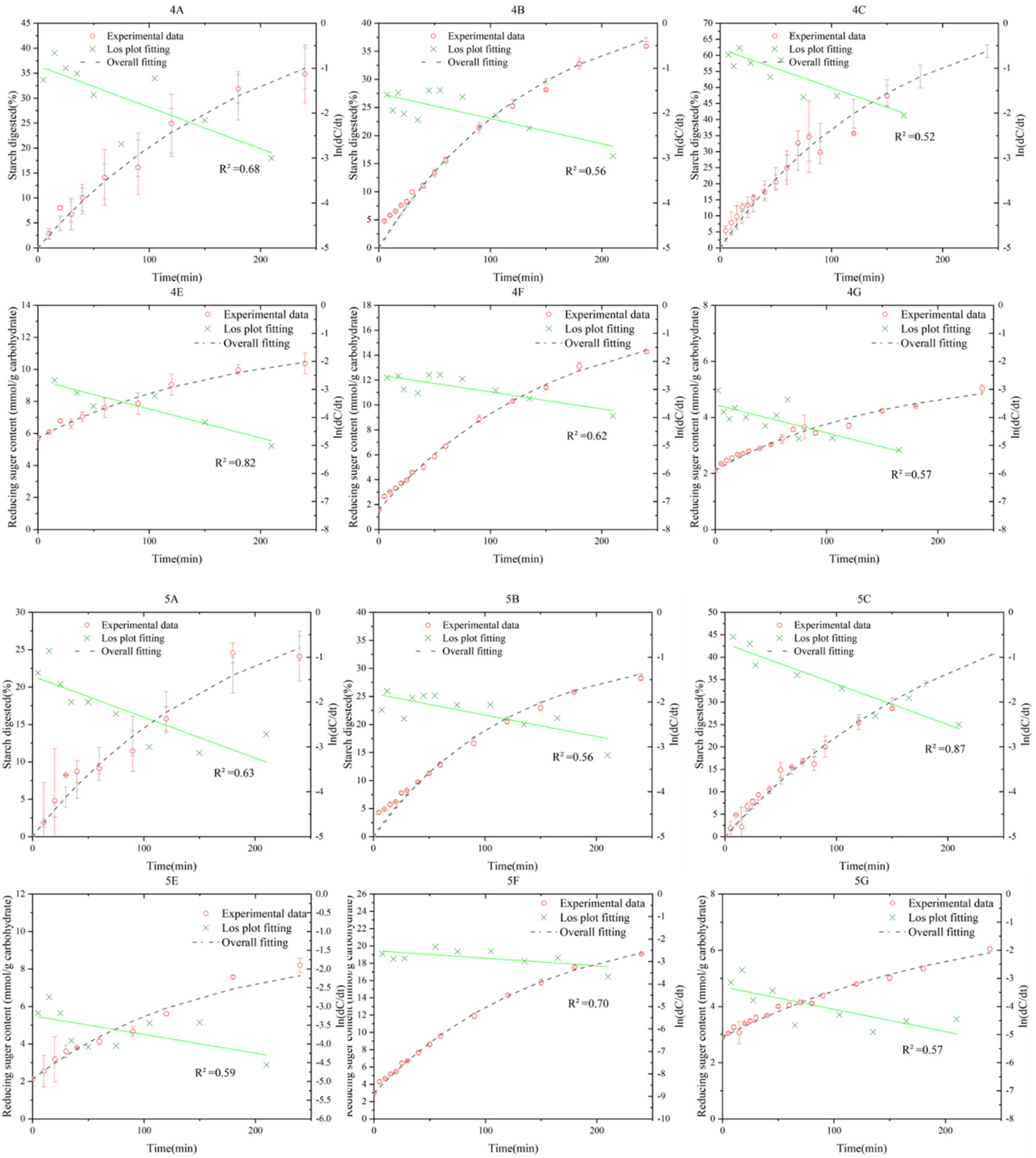
4A
Experimental data
Los plot fitting
Overall fitting
R² =0.68
Starch digested(%)
ln(dC/dt)
Time(min)
4B
Experimental data
Los plot fitting
Overall fitting
R² =0.56
Starch digested(%)
ln(dC/dt)
Time(min)
4C
Experimental data
Los plot fitting
Overall fitting
R² =0.52
Starch digested(%)
ln(dC/dt)
Time(min)
4E
Experimental data
Los plot fitting
Overall fitting
R² =0.82
Reducing suger content (mmol/g carbohydrate)
ln(dC/dt)
Time(min)
4F
Experimental data
Los plot fitting
Overall fitting
R² =0.62
Reducing suger content (mmol/g carbohydrate)
ln(dC/dt)
Time(min)
4G
Experimental data
Los plot fitting
Overall fitting
R² =0.57
Reducing suger content (mmol/g carbohydrate)
ln(dC/dt)
Time(min)
5A
Experimental data
Los plot fitting
Overall fitting
R² =0.63
Starch digested(%)
ln(dC/dt)
Time(min)
5B
Experimental data
Los plot fitting
Overall fitting
R² =0.56
Starch digested(%)
ln(dC/dt)
Time(min)
5C
Experimental data
Los plot fitting
Overall fitting
R² =0.87
Starch digested(%)
ln(dC/dt)
Time(min)
5E
Experimental data
Los plot fitting
Overall fitting
R² =0.59
Reducing suger content (mmol/g carbohydrate)
ln(dC/dt)
Time(min)
5F
Experimental data
Los plot fitting
Overall fitting
R² =0.70
Reducing suger content (mmol/g carbohydrate)
ln(dC/dt)
Time(min)
5G
Experimental data
Los plot fitting
Overall fitting
R² =0.57
Reducing suger content (mmol/g carbohydrate)
ln(dC/dt)
Time(min)

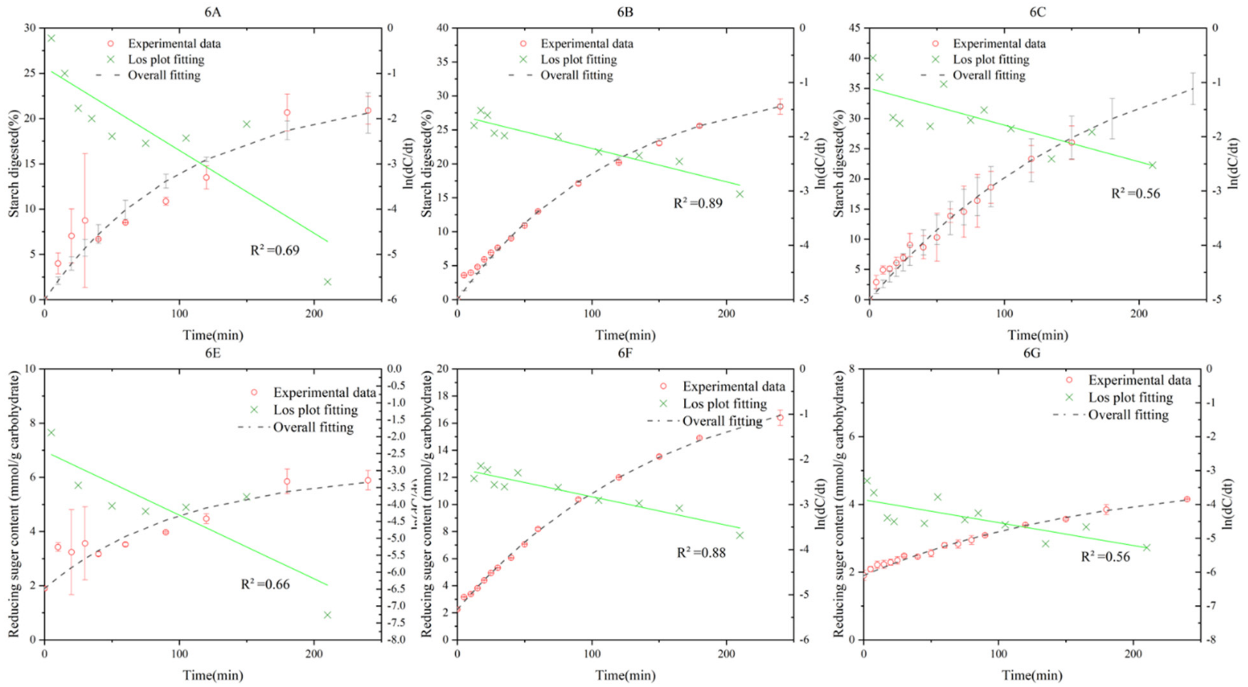
6A
Experimental data
Los plot fitting
Overall fitting
R² =0.69
Starch digested(%)
ln(dC/dt)
Time(min)
6B
Experimental data
Los plot fitting
Overall fitting
R² =0.89
Starch digested(%)
ln(dC/dt)
Time(min)
6C
Experimental data
Los plot fitting
Overall fitting
R² =0.56
Starch digested(%)
ln(dC/dt)
Time(min)
6E
Experimental data
Los plot fitting
Overall fitting
R² =0.66
Reducing suger content (mmol/g carbohydrate)
Time(min)
6F
Experimental data
Los plot fitting
Overall fitting
R² =0.88
Reducing suger content (mmol/g carbohydrate)
ln(dC/dt)
Time(min)
6G
Experimental data
Los plot fitting
Overall fitting
R² =0.56
Reducing suger content (mmol/g carbohydrate)
ln(dC/dt)
Time(min)

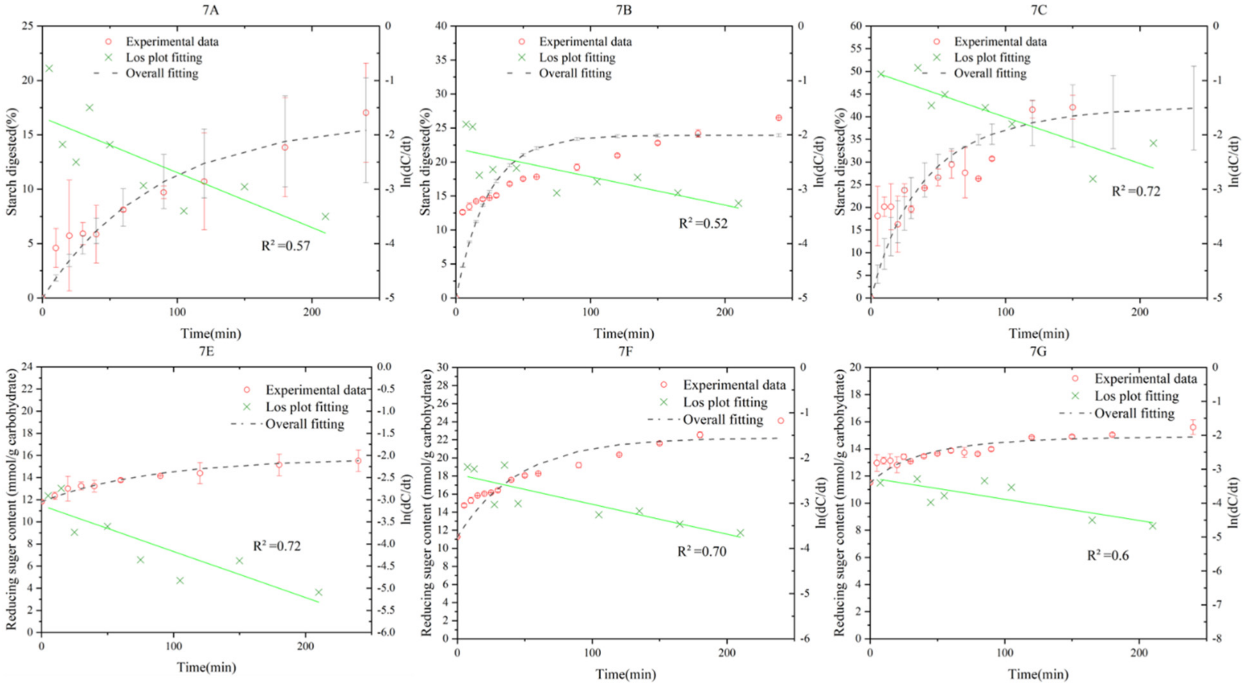
7A
Experimental data
Los plot fitting
Overall fitting
R² =0.57
Starch digested(%)
ln(dC/dt)
Time(min)
7B
Experimental data
Los plot fitting
Overall fitting
R² =0.52
Starch digested(%)
ln(dC/dt)
Time(min)
7C
Experimental data
Los plot fitting
Overall fitting
R² =0.72
Starch digested(%)
ln(dC/dt)
Time(min)
7E
Experimental data
Los plot fitting
Overall fitting
R² =0.72
Reducing suger content (mmol/g carbohydrate)
ln(dC/dt)
Time(min)
7F
Experimental data
Los plot fitting
Overall fitting
R² =0.70
Reducing suger content (mmol/g carbohydrate)
ln(dC/dt)
Time(min)
7G
Experimental data
Los plot fitting
Overall fitting
R² =0.6
Reducing suger content (mmol/g carbohydrate)
ln(dC/dt)
Time(min)

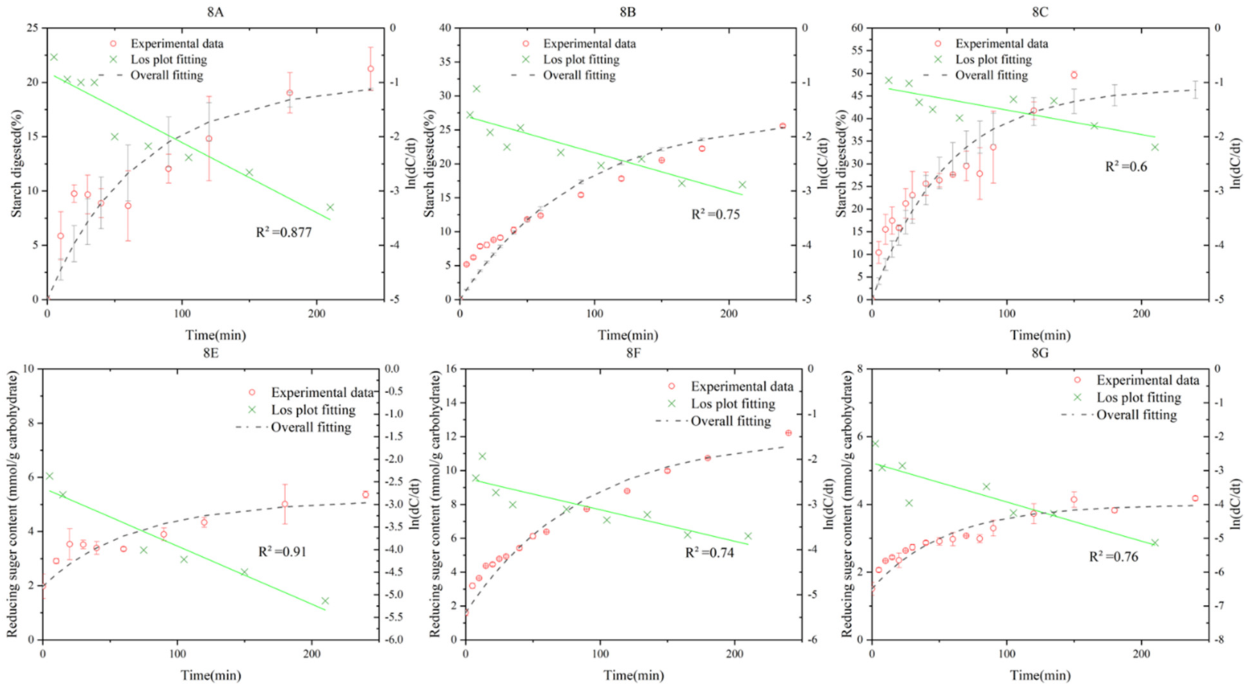
8A
Experimental data
Los plot fitting
Overall fitting
R² =0.877
Starch digested(%)
ln(dC/dt)
Time(min)
8B
Experimental data
Los plot fitting
Overall fitting
R² =0.75
Starch digested(%)
ln(dC/dt)
Time(min)
8C
Experimental data
Los plot fitting
Overall fitting
R² =0.6
Starch digested(%)
ln(dC/dt)
Time(min)
8E
Experimental data
Los plot fitting
Overall fitting
R² =0.91
Reducing suger content (mmol/g carbohydrate)
ln(dC/dt)
Time(min)
8F
Experimental data
Los plot fitting
Overall fitting
R² =0.74
Reducing suger content (mmol/g carbohydrate)
ln(dC/dt)
Time(min)
8G
Experimental data
Los plot fitting
Overall fitting
R² =0.76
Reducing suger content (mmol/g carbohydrate)
ln(dC/dt)
Time(min)

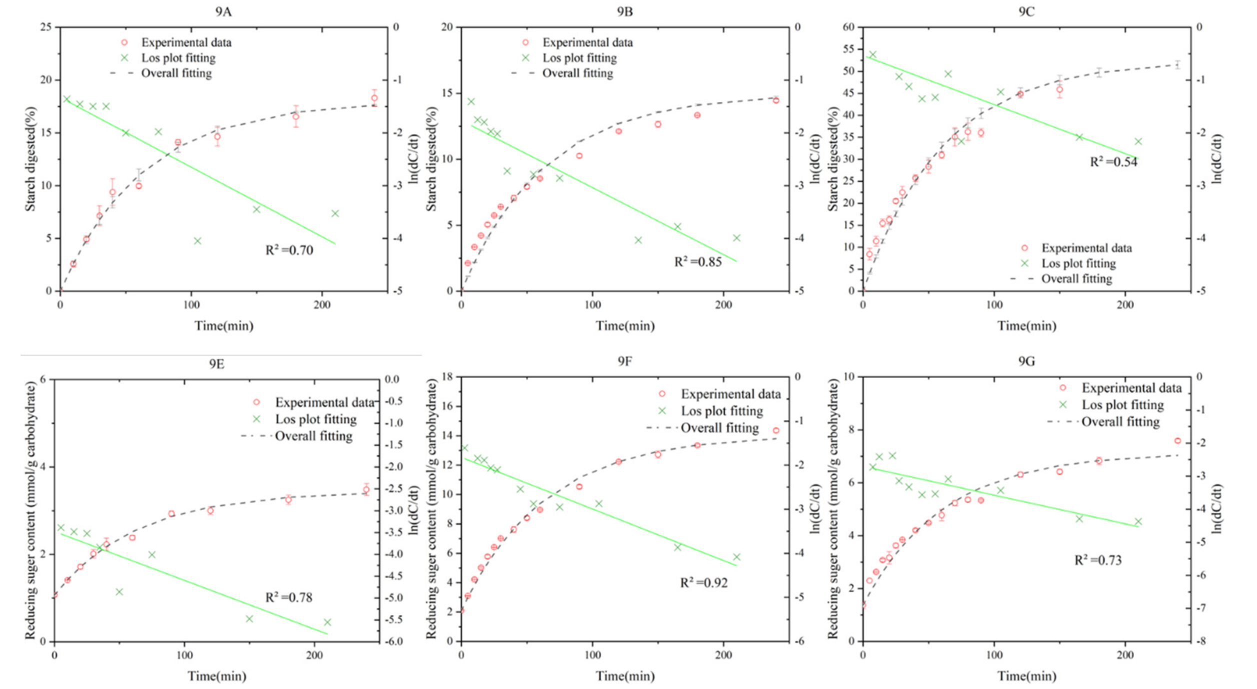
9A
Experimental data
Los plot fitting
Overall fitting
R² =0.70
Starch digested(%)
ln(dC/dt)
Time(min)
9B
Experimental data
Los plot fitting
Overall fitting
R² =0.85
Starch digested(%)
ln(dC/dt)
Time(min)
9C
Experimental data
Los plot fitting
Overall fitting
R² =0.54
Starch digested(%)
ln(dC/dt)
Time(min)
9E
Experimental data
Los plot fitting
Overall fitting
R² =0.78
Reducing suger content (mmol/g carbohydrate)
ln(dC/dt)
Time(min)
9F
Experimental data
Los plot fitting
Overall fitting
R² =0.92
Reducing suger content (mmol/g carbohydrate)
ln(dC/dt)
Time(min)
9G
Experimental data
Los plot fitting
Overall fitting
R² =0.73
Reducing suger content (mmol/g carbohydrate)
ln(dC/dt)
Time(min)

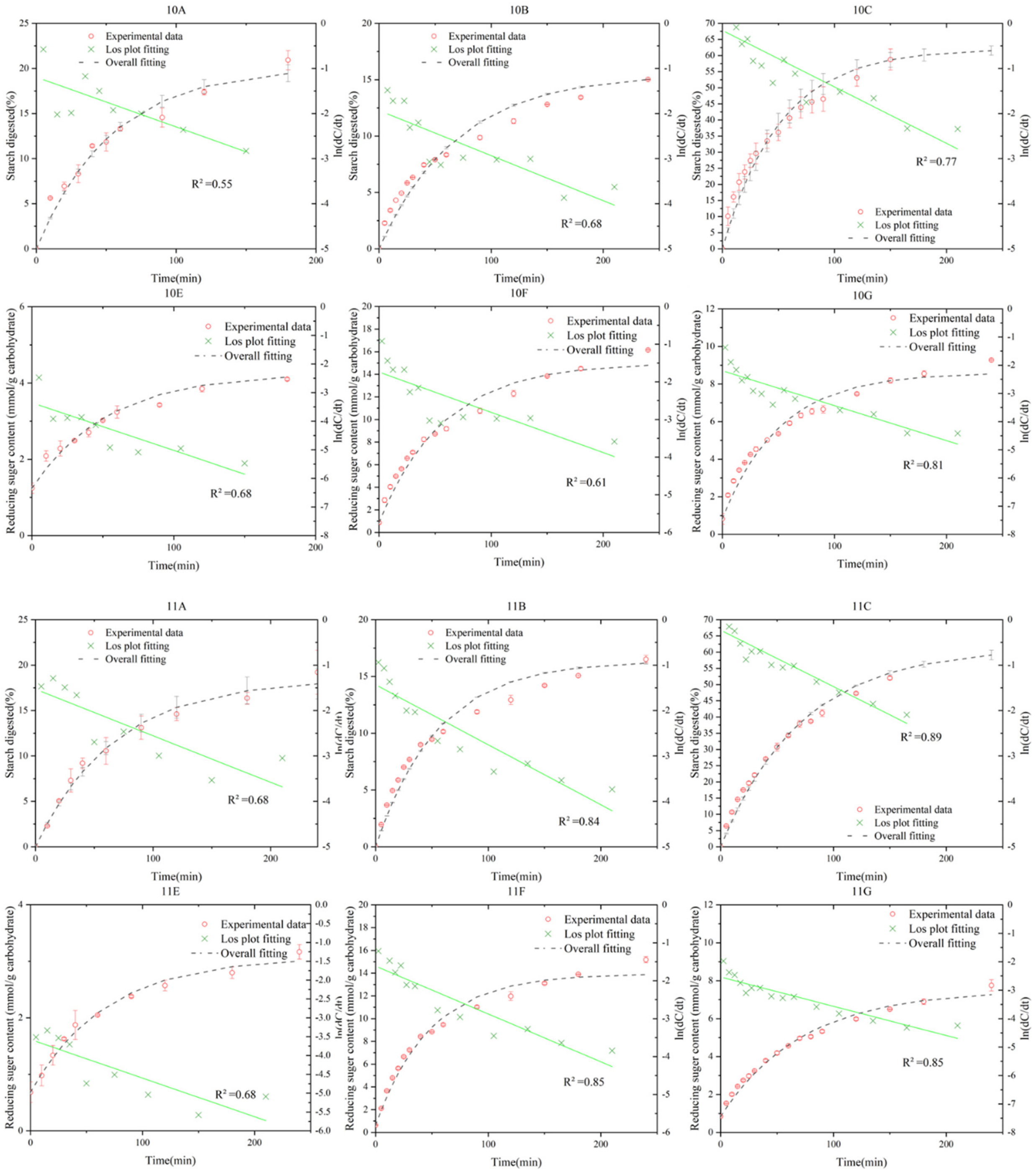
10A
Experimental data
Los plot fitting
Overall fitting
R² =0.55
Starch digested(%)
ln(dC/dt)
Time(min)
10B
Experimental data
Los plot fitting
Overall fitting
R² =0.68
Starch digested(%)
ln(dC/dt)
Time(min)
10C
Experimental data
Los plot fitting
Overall fitting
R² =0.77
Starch digested(%)
ln(dC/dt)
Time(min)
10E
Experimental data
Los plot fitting
Overall fitting
R² =0.68
Reducing suger content (mmol/g carbohydrate)
ln(dC/dt)
Time(min)
10F
Experimental data
Los plot fitting
Overall fitting
R² =0.61
Reducing suger content (mmol/g carbohydrate)
ln(dC/dt)
Time(min)
10G
Experimental data
Los plot fitting
Overall fitting
R² =0.81
Reducing suger content (mmol/g carbohydrate)
ln(dC/dt)
Time(min)
11A
Experimental data
Los plot fitting
Overall fitting
R² =0.68
Starch digested(%)
Time(min)
11B
Experimental data
Los plot fitting
Overall fitting
R² =0.84
Starch digested(%)
Time(min)
11C
Experimental data
Los plot fitting
Overall fitting
R² =0.89
Starch digested(%)
ln(dC/dt)
Time(min)
11E
Experimental data
Los plot fitting
Overall fitting
R² =0.68
Reducing suger content (mmol/g carbohydrate)
Time(min)
11F
Experimental data
Los plot fitting
Overall fitting
R² =0.85
Reducing suger content (mmol/g carbohydrate)
ln(dC/dt)
Time(min)
11G
Experimental data
Los plot fitting
Overall fitting
R² =0.85
Reducing suger content (mmol/g carbohydrate)
ln(dC/dt)
Time(min)

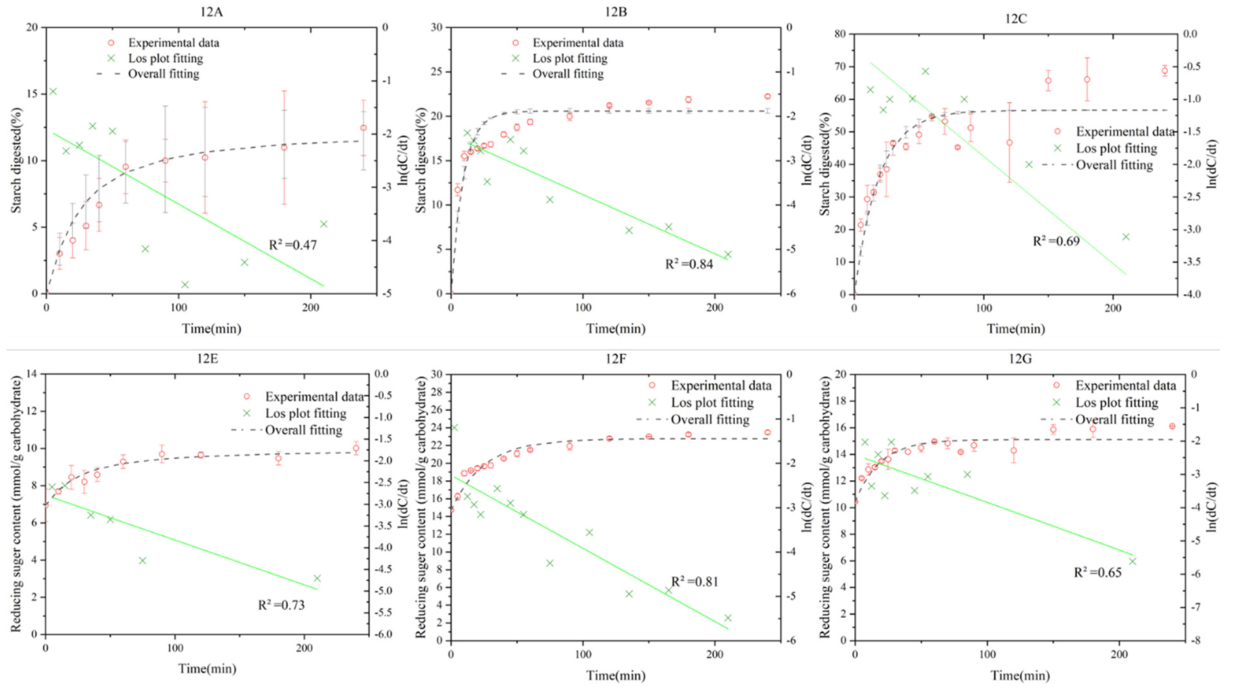
12A
Experimental data
Los plot fitting
Overall fitting
R² =0.47
Starch digested(%)
ln(dC/dt)
Time(min)
12B
Experimental data
Los plot fitting
Overall fitting
R² =0.84
Starch digested(%)
ln(dC/dt)
Time(min)
12C
Experimental data
Los plot fitting
Overall fitting
R² =0.69
Starch digested(%)
ln(dC/dt)
Time(min)
12E
Experimental data
Los plot fitting
Overall fitting
R² =0.73
Reducing suger content (mmol/g carbohydrate)
ln(dC/dt)
Time(min)
12F
Experimental data
Los plot fitting
Overall fitting
R² =0.81
Reducing suger content (mmol/g carbohydrate)
ln(dC/dt)
Time(min)
12G
Experimental data
Los plot fitting
Overall fitting
R² =0.65
Reducing suger content (mmol/g carbohydrate)
ln(dC/dt)
Time(min)

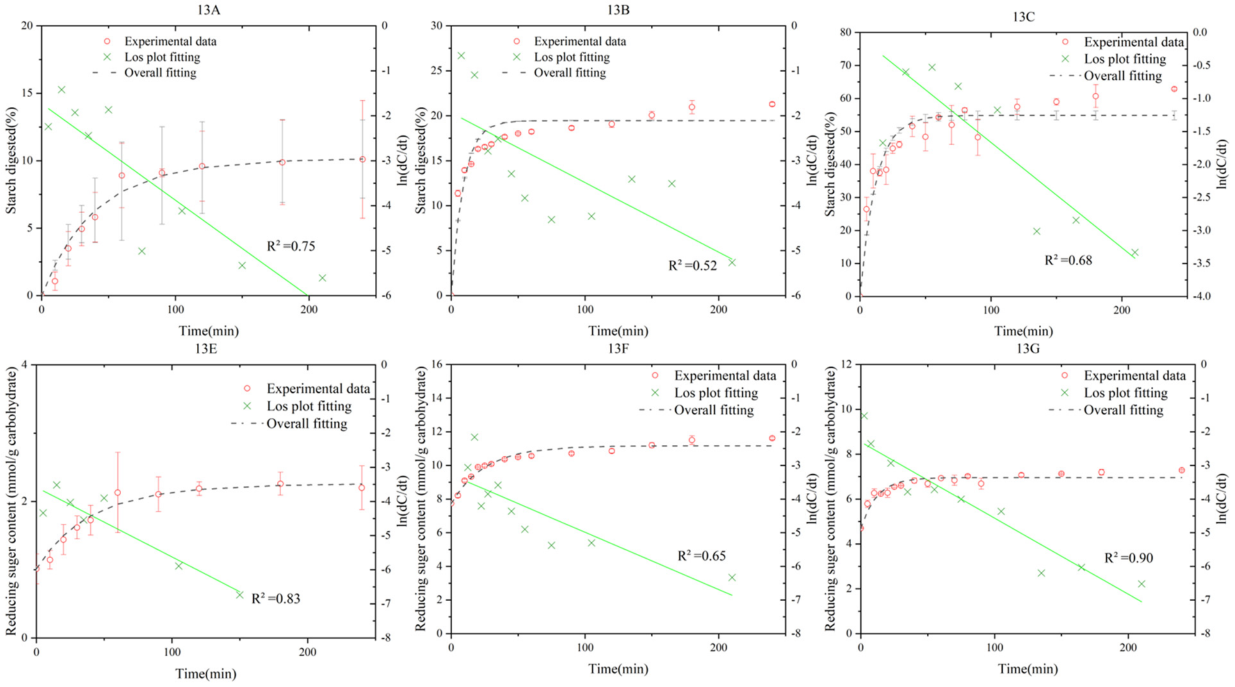
13A
Experimental data
Los plot fitting
Overall fitting
R² =0.75
Starch digested(%)
ln(dC/dt)
Time(min)
13B
Experimental data
Los plot fitting
Overall fitting
R² =0.52
Starch digested(%)
ln(dC/dt)
Time(min)
13C
Experimental data
Los plot fitting
Overall fitting
R² =0.68
Starch digested(%)
ln(dC/dt)
Time(min)
13E
Experimental data
Los plot fitting
Overall fitting
R² =0.83
Reducing suger content (mmol/g carbohydrate)
ln(dC/dt)
Time(min)
13F
Experimental data
Los plot fitting
Overall fitting
R² =0.65
Reducing suger content (mmol/g carbohydrate)
ln(dC/dt)
Time(min)
13G
Experimental data
Los plot fitting
Overall fitting
R² =0.90
Reducing suger content (mmol/g carbohydrate)
ln(dC/dt)
Time(min)

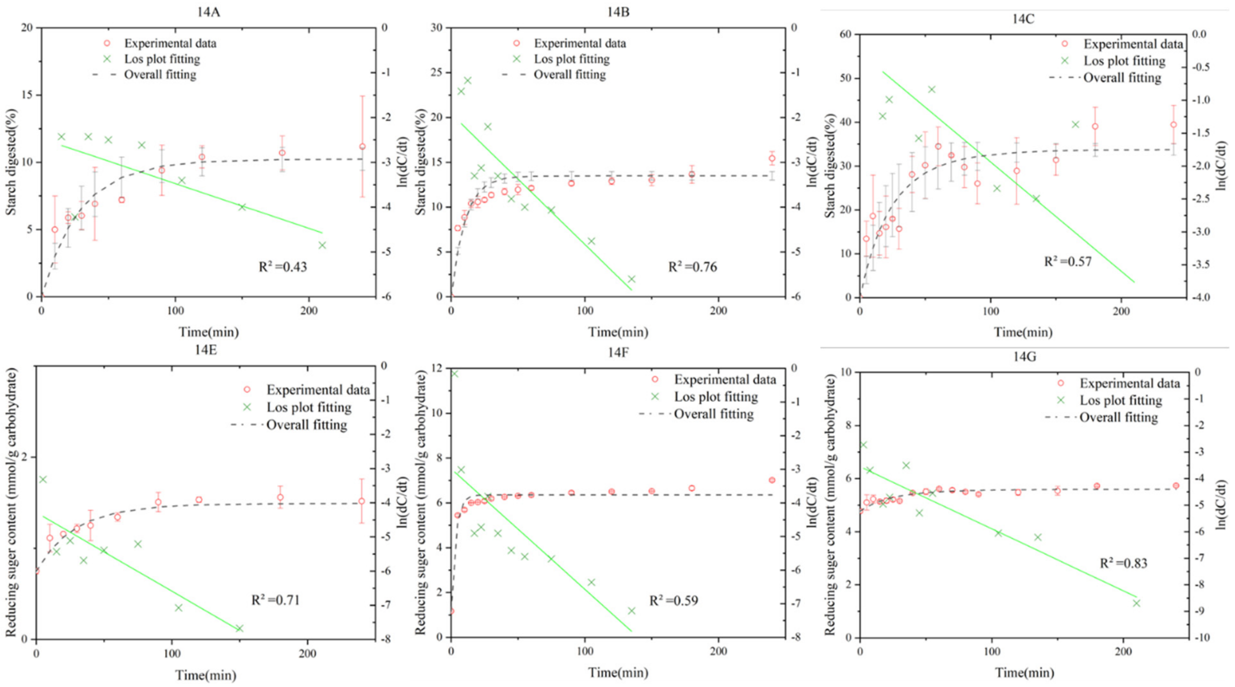

Figure S1. LOS plot for all samples (2-14) when digested using the INFOGEST protocol (A) and the single enzyme digestion model using porcine pancreatin (B) or pure α-amylase (C), showing only one digestion phase. The upper pane (A, B & C) was the fitting results in terms of the digestion results of starch digested (%) as a function of the digestion time (min), while the lower pane （E ,F & G）was the fitting results in terms of the digestion results of the total amount of released reducing sugar content (mmol) normalized to the total carbohydrates as a function of the digestion time (min).

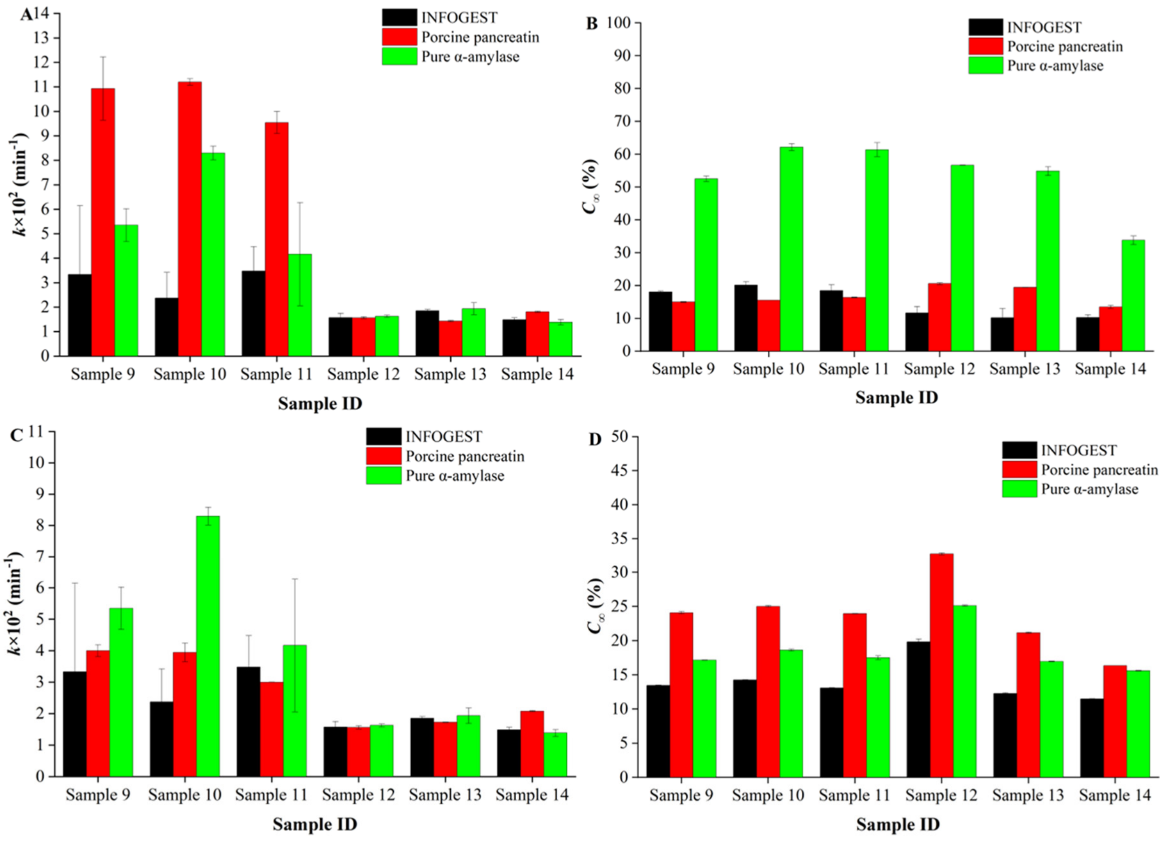

Figure S2. The parameters of samples 9-12 were obtained by fitting the in vitro digestion diagram with a single first-order kinetics (SK) model. (A) and (B) are the fitting results of the total percentage of starch digested, (C) and (D) are the fitting results of the reducing sugar content which has been normalized to the total weight of car-bohydrate. Data was based on duplicate measurements.
